# Immunomodulatory Effects of Herbal Compounds Quercetin and Curcumin on Cellular and Molecular Functions of Bovine-Milk-Isolated Neutrophils toward *Streptococcus agalactiae* Infection

**DOI:** 10.3390/ani11113286

**Published:** 2021-11-17

**Authors:** Purichaya Disbanchong, Wichayaporn Punmanee, Anyaphat Srithanasuwan, Noppason Pangprasit, Kanruethai Wongsawan, Witaya Suriyasathaporn, Phongsakorn Chuammitri

**Affiliations:** 1 Department of Veterinary Biosciences and Public Health, Faculty of Veterinary Medicine, Chiang Mai University, Chiang Mai 50100, Thailand; purichaya.dis@gmail.com (P.D.); wichayaporn.punmanee@gmail.com (W.P.); kanruethai.w@cmu.ac.th (K.W.); 2 Department of Food Animal Clinics, Faculty of Veterinary Medicine, Chiang Mai University, Chiang Mai 50100, Thailand; numwan.sw@gmail.com (A.S.); witaya.s@cmu.ac.th (W.S.); 3 Research Center of Producing and Development of Products and Innovations for Animal Health and Production, Chiang Mai University, Chiang Mai 50100, Thailand; 4 Akkhraratchakumari Veterinary College, Walailak University, Nakhon Si Thammarat 80160, Thailand; panny.2.yaa@gmail.com

**Keywords:** quercetin, curcumin, neutrophil, *Streptococcus agalactiae*, mastitis, cow

## Abstract

**Simple Summary:**

Many herbal remedies contain quercetin or curcumin as one of the active ingredients. The broad pharmacologic effects of these herbal compounds have received significant attention in recent years for their roles in the modulation of the innate immune response in humans and animals. The use of quercetin and curcumin in dairy cattle may be the most promising alternative to control *S. agalactiae*, which are prominent pathogens involved in bovine mastitis. However, the mechanisms by which quercetin and curcumin facilitate the elimination of invading pathogens is not yet fully understood. This study examined the cellular and molecular levels of the innate immune activities induced by quercetin and curcumin, separately, in milk-isolated bovine neutrophils during *S. agalactiae* stimulation. Our results demonstrate that quercetin and curcumin present beneficial effects, including increasing cell migration and antioxidant activities, enhancing phagocytosis and bacterial killing, increasing NET release, altered patterns of gene expression, and manipulating cell death. Our results regarding these two herbal compounds indicate that they may alleviate inflammation due to the innate immune cell dynamic in bacterial mastitis. Further investigations are needed to confirm our observations and examine the underlying mechanisms.

**Abstract:**

Herbal phytochemicals featuring active ingredients including quercetin and curcumin have shown potential in treating human and animal diseases. The current study investigated their potential function in vitro for host immunomodulation associated with *Streptococcus agalactiae* subclinical bovine mastitis via milk-isolated neutrophils. Our results showed a positive influence on cellular migration, reactive oxygen species (ROS) generation, phagocytosis, and bacterial killing as well as neutrophil extracellular traps (NETs) release. This study also highlighted several important molecular aspects of quercetin and curcumin in milk-isolated neutrophils. Gene expression analyses by RT-PCR revealed significant changes in the expression of proinflammatory cytokines (*IL1B*, *IL6*, and *TNF*), ROS (*CYBA*), phagocytosis (*LAMP1*), and migration (*RAC*). The expression levels of apoptotic genes or proteins in either pro-apoptosis (*CASP3* and *FAS*) or anti-apoptosis (*BCL2*, *BCL2L1*, and *CFLAR*) were significantly manipulated by the effects of either quercetin or curcumin. A principal component analysis (PCA) identified the superior benefit of quercetin supplementation for increasing both cellular and molecular functions in combating bacterial mastitis. Altogether, this study showed the existing and potential benefits of these test compounds; however, they should be explored further via in vivo studies.

## 1. Introduction

Bovine mastitis is the most significant problem in the dairy industry globally. This inflammation-driven disease of the bovine mammary glands causes a substantial financial burden in the dairy sector. Bovine mastitis is caused by infection as a result of pathogenic bacteria, such as *Staphylococcus aureus*, *Streptococcus agalactiae*, and *Escherichia coli*. Streptococci are common etiological agents of bovine mastitis [1].

The polymorphonuclear neutrophil leukocytes (PMN) and macrophages are abundantly found in bovine mammary glands as the primary innate immune phagocytes [2]. The infiltration of the innate immune cells, occurring at an early stage, into the mammary tissues and the cytokines released by these cells are critical for eliciting defense responses in bovine mastitis. The functionally essential intracellular or extracellular duties of cells, i.e., neutrophil extracellular traps (NETs), are performed by mammary PMNs, allowing for more efficient internalization of invading and killing microbes [3,4].

Used alone or in combination with others, certain compounds of herbal origin, such as flavonoids, quinones, polyphenols, and carotenoids, are a valuable source of bioactive ingredients. They have shown promise as either alternative or complementary supplements in treating and preventing many diseases [5,6]. Quercetin and curcumin have many biological and therapeutic properties including anti-inflammatory, antineoplastic, antioxidant, antidiabetic, antihypertensive activity, and wound healing [5,6,7,8,9]. We have previously highlighted that quercetin, the bioflavonoids found in fruits, vegetables, leaves, and grains, has protective potential and prophylactic possibilities in circulating bovine neutrophils against *Escherichia coli* [3,4]. From a resource perspective, both quercetin and curcumin exhibit similar modes of action that could enable and promote immune responses. These plant compounds have been extensively studied and used for their immunomodulatory roles in humans and animals [5,8,10,11,12].

The application of quercetin and curcumin has been anticipated as an alternative to control bovine mastitis, but the cellular and molecular mechanisms by which these two compounds help ameliorate the severity of bovine mastitis are not yet fully understood. Hence, this study aimed to investigate the immunomodulatory effects of quercetin and curcumin in activated milk PMNs during the in vitro challenge of *Streptococcus agalactiae*.

## 2. Materials and Methods

### 2.1. Milk Samples Collection and Milk PMN Isolation

The study was conducted in two smallholder dairy herds in the Mae-Wang Dairy Cooperative in Chiang Mai, Thailand. The farms had a high bulk-milk somatic cell count (SCC, >1,000,000 cells/mL) from a herd mastitis investigation. All quarter milk samples were tested for the presence of subclinical mastitis via screening based on a California mastitis test (CMT) score of 2 or SCC of 500,000–1,000,000 cells/mL. All affected quarters (*n* = 20) were sampled aseptically for subsequent bacteriological culture, PCR, and milk PMN isolation. Collection of subclinical quarter milk samples of milking cows was performed only once. In terms of limitations, we used only 20 quarter milk samples to conduct our experiments. The sample size was selected based on our previous experience and appropriate estimates of what would be feasible for variation within the immune cell responses.

The animal study protocol was reviewed and approved by the Faculty of Veterinary Medicine at Chiang Mai University, Animal Care and Use Committee (FVM-ACUC) Ref. No. S21/2563. Two tubes of fifteen (15) mL of quarter milk sample were collected for immediate milk PMN isolation. Quarter milk was centrifuged at 1000× *g* for 10 min, 4 °C (Allegra X-15R Centrifuge, Beckman Coulter, Brea, CA, USA), followed by the discarding of cream and whey from the sample. The remaining cell pellet was washed twice with PBS, centrifuged at 700× *g* for 5 min, and then resuspended in cold RPMI-1640 medium (Thermo Fisher Scientific, Waltham, MA, USA) supplemented with 1% heat-inactivated fetal bovine serum (FBS, Thermo Fisher Scientific, Waltham, MA, USA). The isolated milk PMNs in each quarter were divided into the control group (PBS) and two treatment groups (i.e., quercetin and curcumin). Viability was assessed by trypan blue dye exclusion (Thermo Fisher Scientific, Waltham, MA, USA). Finally, cell density was adjusted to approximately 3 × 10^6^ cells per mL. Freshly isolated milk PMNs were seeded onto circular coverslips (15 mm diameter) that were placed into a 24-well plate to make cytospin slides. The plate was centrifuged at 1200 rpm for 3 min. Slides were fixed with absolute ethanol, stained with Dip Quick, and scanned with a Pannoramic MIDI slide scanner (3D HISTECH, Budapest, Hungary).

### 2.2. Bacterial Growth Condition, Fluorescent Labeling, and Opsonization

*Streptococcus agalactiae* used throughout the experiment was originally from field isolates and stored at −20 °C in glycerol/Tryptic soy broth (TSB, HIMEDIA, Mumbai, India). The clinical history of the *S. agalactiae* isolates used in this study was field isolates from subclinical milk quarters with mild-to-moderate SCC where the farm was located. Before being used in the experiment, an aliquot of bacteria from the frozen collection was thawed and was inoculated onto Tryptic soy agar (TSA, HIMEDIA, Mumbai, India) plates with 5% bovine blood and grown overnight (24 h) at 37 °C. In the experiment, *S. agalactiae* was freshly prepared by the colony-picking method and cultured in TSB at 37 °C in an incubator for 16 h. The bacteria number was adjusted to approximately 10^8^ CFU/mL before use in the experiment.

Live *S. agalactiae* was grown to a log phase, suspended in a PBS solution, and the heat-killed at 70 °C for 60 min [4]. Heat-killed bacteria were resuspended at a density of 10^8^ CFU/mL in 1 µg/mL goat anti-mouse IgG (H + L) antibody, Alexa Fluor^®^ 488 (Invitrogen, Thermo Fisher Scientific, Waltham, MA, USA). Bacteria were fluorescently labeled for 30 min at 4 °C. Afterwards, *S. agalactiae* was washed extensively with PBS to remove the free dye, adjusted to 10^7^ CFU/mL with PBS, and stored at 4 °C until use. Fluorescently labeled *S. agalactiae* was opsonized with 10% heat-inactivated normal bovine serum for 20 min at 37 °C before being used in phagocytosis assay.

### 2.3. Quercetin

Quercetin hydrate (QH) with a purity of ≥95% by HPLC and containing ≥2.9% water as determined by Karl Fischer was used (Sigma-Aldrich, St. Louis, MO, USA). Details regarding the preparation of the stock quercetin solution (5 mM) and working solution (50 μM) are described as follows. The stock solution was prepared by dissolving dry quercetin powder in 95% ethanol and sterile filter. A working solution was made by diluting the stock 1:100 with PBS. The working solution was wrapped with foil to protect it from light. All solutions were freshly prepared on the day of the experiment and stored at room temperature until use. This concentration was previously determined to be safe to use for the stimulation of bovine neutrophils [3,4,13].

### 2.4. Curcumin

Curcumin is the major constituent of turmeric powder. The percentage of curcumin in the turmeric crude extract was analyzed using high-performance liquid chromatography (HPLC), as described previously [14]. The preparation of the stock curcumin solution (6.5 mM) and working solution (65 μM) are initially described as follows. Dry powder was dissolved in methanol and sterile filter to make a 6.5 mM stock solution (15 mg powder in 1 mL methanol). Working curcumin solution was made by diluting the stock 1:100 with PBS. The working solution was wrapped with foil to protect it from light.

### 2.5. Viability Detection of Streptococcus agalactiae Treated with Different Concentrations of Quercetin or Curcumin via Agar Gel Diffusion Assay

An in vitro assay of antibacterial activity of quercetin/curcumin was performed as previously described with some modifications [15]. An aliquot of *Streptococcus agalactiae* was washed, diluted to 0.5 McFarland, plated on Mueller Hinton Agar (MHA). The agar diffusion method was performed by using sterile Whatman filter paper punched out to 6 mm disks. The disks were dipped in different concentrations of quercetin (12, 25, 50, and 100 μM) or curcumin (32, 65, 163, and 325 μM) and penicillin G antimicrobial susceptibility disk (Thermo Fisher Scientific, Waltham, MA, USA) for 1 min. The disks were placed on the spread plates prepared with *Streptococcus agalactiae* and pressed down to ensure complete, even contact with the bacteria, as depicted in Figure 2A. The plate was incubated at 37 °C for 16 h. Image capture was documented for further analysis using a GelMax Imager (Ultra-Violet Products, Cambridge, UK).

### 2.6. In Vitro Cytotoxicity Assay of Different Concentrations of Quercetin/Curcumin on Milk PMNs via MTT Assay

A total of 1 × 10^5^ isolated milk PMNs were seeded into a 96-well flat-bottom plate in duplicate and later incubated with PBS or serial dilutions of quercetin hydrate (13, 25, 50, and 100 μM) or curcumin (32, 65, 163, and 325 μM) in RPMI-1640 medium at 37 °C with 5% CO_2_ for 45 min. After incubation, the plate was spun at 1200 rpm for 3 min (LMC-3000, BioSan, Riga, Latvia) and then the supernatant was discarded. All wells were infused with 2 μg/mL 3-[4,5-dimethylthiazole-2-yl]-2,5-diphenyltetrazolium bromide (MTT, Sigma-Aldrich, St. Louis, MO, USA) in PBS [3]. After 15 min incubation, optical density (OD) of colored formazan was measured at OD_570_ using an automated microplate reader (Anthos Labtec Instruments, Wals, Austria). Percentage of cell viability was quantified by the following equation: % viable cells = (OD _sample_ − OD _blank_) × 100 (OD _control_ − OD _blank_)

### 2.7. In Vitro Quercetin or Curcumin Treatment of Isolated Milk PMNs

To analyze the modulator effects of quercetin or curcumin on milk PMNs, cell stimulation was performed. Freshly isolated milk PMNs were seeded at 3 × 10^5^ cells per well (for most assays) into duplicate 96-well flat tissue culture plates. Cells were treated with either 50 μM quercetin or 65 μM curcumin for 30 min at 37 °C with 5% CO_2_ or treated with PBS, the latter of which served as the control [3]. After treatment, milk PMNs were washed once with PBS and harvested by centrifugation. For gene expression study and Western blot, 3 × 10^6^ cells were dispended into microcentrifuge tubes and stimulated as stated earlier. Milk PMN pellets were resuspended in RNAlater (Thermo Fisher Scientific, Waltham, MA, USA), as per the manufacturer’s instructions, to preserve RNA for further analysis or kept frozen (−80 °C) until ready for protein extraction.

### 2.8. Measurement of Intracellular Reactive Oxygen Species (ROS)

For ROS examination experiments, quercetin- and curcumin-treated milk PMNs were activated to produce ROS with *S. agalactiae* (MOI of 10) in PBS w/Ca^2+^/Mg^2+^. The cells were then incubated for 30 min at 37 °C with 5% CO_2_. Then the cells were washed with PBS and centrifuged at 1200 rpm for 3 min, and the supernatant was discarded. Then 10 µM H_2_DCF-DA (Thermo Fisher Scientific, Waltham, MA, USA) was loaded into each well to stain the intracellular H_2_O_2_ [3]. Cells were incubated in the dark for 15 min, then washed with cold Hanks’ balanced salt solution (HBBS, Thermo Fisher Scientific, Waltham, MA, USA), and sample acquisition (10,000 events) was performed on ROS-containing cells using a DxFLEX Flow Cytometer (Beckman Coulter, Brea, CA, USA) and analyzed by FlowJo 10 (Treestar, Ashland, OR, USA) [16].

### 2.9. Phagocytosis

The phagocytosis of *S. agalactiae* was assessed via flow cytometry. Treated cells (3 × 10^5^ cells) were mixed with opsonized fluorescently labeled *S. agalactiae* (MOI of 10) in duplicate wells of a 96-well, flat-bottom cell culture plate. To promote the uptake of *S. agalactiae*, the cell mixture was centrifuged at 1200 rpm, 3 min, and the milk PMNs were allowed to internalize the bacteria for 45 min at 37 °C, 5% CO_2_ [4]. After incubation, cells were washed extensively with ice-cold PBS, and sample acquisitions (10,000 events) were acquired on DxFLEX Flow Cytometer and analyzed by FlowJo software [16].

### 2.10. Bacterial Killing (MTT) Assay and Spot Dilution Assay

Milk PMN’s bactericidal ability was evaluated using a semi-quantitative MTT assay to indicate the percentage of bacterial viability [3]. We also carried out a qualitative method of bacterial colony scoring (spot plate assay) after killing assay, as previously described with modifications [15].

*Streptococcus agalactiae* were freshly propagated as described in the previous section. Live bacteria were opsonized with normal bovine serum and diluted to a final concentration of 1 × 10^7^ CFU/mL. Separately, quercetin- and curcumin-treated cells (3 × 10^5^ cells) were loaded into duplicate wells of a 96-well plate and, subsequently, opsonized bacteria were added at a 1:10 ratio. The plate was centrifuged (1200 rpm, 3 min) and placed in an incubator for 45 min. After incubation, the plate was again centrifuged to remove non-ingested bacteria. Hypotonic solution (diH_2_O) was used for releasing internalized bacteria from milk PMNs (5 min at RT). After lysing, all wells were supplemented with Mueller Hinton (MH) broth with 2 μg/mL MTT. The plate was incubated for a total of 90 min at 37 °C. The MTT-insoluble formazan was solubilized to colored crystals by adding dimethyl sulfoxide (DMSO). Colorimetric detection was done at a wavelength of 570 nm. In each experiment, OD from MTT solution only (Blank) was included to indicate no live bacteria were present. Percentage of bacterial killing was calculated by substituting measured OD values into the following formula: % of killing = 100 − [(OD_sample_ − OD_Blank_) × 100]

Spot dilution assays were performed by an aliquot of 2 μL of lysed milk PMNs from earlier steps in the MTT assay (control, quercetin-treated, and curcumin-treated milk PMNs). A series of ten-fold dilutions (2 μL of each 10^−1^ to 10^−5^) was spotted on Tryptic soy agar (TSA) plates with 5% bovine blood and grown overnight (24 h) at 37 °C. Image capture was documented for comparison of colony sizes by using the GelMax Imager.

### 2.11. Transwell In Vitro Migration Assay

Directed milk PMN migration toward live *Streptococcus agalactiae* was studied using Transwell cell migration chambers. The lower chambers of the 24-well Transwell plate were dispensed with 600 μL of cell culture media alone (RPMI-1640) or live *S. agalactiae* (3 × 10^5^ bacteria). Then the polycarbonate membrane Transwell inserts (8 μm of pore size, Corning, Corning, NY, USA) were placed over the upper wells. Stimulated cells (in 100 μL volume) were added to Transwell inserts. Plates were incubated at 37 °C in an incubator with 5% CO_2_ for 45 min. After incubation, filter inserts were dislodged, and the liquid portion containing migrated cells of the lower part of the wells was collected for further analysis. The counting of migrated cells was acquired using the forward (FSC) and side scatter (SSC) of a flow cytometer, according to a previously reported method [15].

### 2.12. Quantification and Visualization of Neutrophil Extracellular Trap (NET) Release of Milk PMNs

Stimulated milk PMNs (3 × 10^5^ cells) were seeded into duplicate wells of a 96-well plate. Cells stimulated with PBS served as controls. Live *Streptococcus agalactiae* (3 × 10^6^ bacteria) was added to all wells. Additionally, all wells were also supplemented with PBS with Ca_2_^+^ and Mg_2_^+^ before incubation at 37 °C, 5% CO_2_ for 150 min. After activation, plates were centrifuged at 1200 rpm for 3 min, and the supernatant was discarded. Ice-cold RPMI 1640 media was added to each well and gently mixed by pipetting and centrifuge. The supernatant containing extracellular DNAs was transferred to new plates. NET-DNA was quantified using a fluorescent dye (Hoechst 33342, Thermo Fisher Scientific, Waltham, MA, USA) at 5 mg/mL [15]. Fluorescence measurement of stained NETs was measured with a Synergy^™^ HT Multi-Detection Microplate Reader using an excitation wavelength at 360 nm and emission at 470 nm. The relative fluorescence units (RFU) were recorded [16].

NET structures were also confirmed by examination under fluorescent microscopy by staining the NET structure with Hoechst 33342 (nuclei) and H_2_DCF-DA for ROS, as described in a previous method [15]. In brief, 8-well chamber slides (SPL Life Sciences, Gyeonggi-do, Korea) were filled with either stimulated milk PMNs (1 × 10^5^ cells) or PBS stimulation, which served as controls. Live *S. agalactiae* (5 × 10^5^ bacteria) and PBS with Ca_2_^+^ and Mg_2_^+^ were added to all wells. Cells were left stimulation for 180 min at 37 °C, 5% CO_2_. All samples were rinsed with ice-cold PBS and fixed with cold 4% paraformaldehyde (PFA) for 15 min. The slides were rinsed with ice-cold PBS and stained with 1:60 dilution of Hoechst 33342, 10 mg/mL solution plus 10 µM final concentration of H_2_DCF-DA for 10 min in the dark and rinsed. The chamber slides were disassembled and a drop of ProLong™ Glass Antifade Mountant (Invitrogen, Thermo Fisher Scientific, Waltham, MA, USA) was applied. The visualization and image capture were performed with an Axio Scope A1 Fluorescence Microscope (Carl Zeiss, Thornwood, NY, USA) at 10× and 20× objectives.

### 2.13. Quantitative Real-Time PCR (qPCR)

To explore the effects of quercetin/curcumin on milk PMN gene expression after encountering *S. agalactiae* for 1 h, preserved RNAs were extracted using RNAzol^®^ RT (Sigma-Aldrich, St. Louis, MO, USA) following the manufacturer’s instructions [15]. The cDNAs were synthesized using 2 µg of total RNA by Tetro cDNA Synthesis Kit (Bioline, Taunton, MA, USA), and 100 ng samples of cDNA from milk PMNs were quantitatively analyzed in triplicate for the mRNA transcripts of interleukin 1 beta (*IL1B*), interleukin 6 (*IL6*), tumor necrosis factor (*TNF*), cytochrome b-245 alpha chain (*CYBA*, also called p22^phox^), lysosomal associated membrane protein 1 (*LAMP1*), Ras-related C3 botulinum toxin substrate (*RAC*), B-cell CLL/lymphoma 2 (*BCL2*), BCL2 like 1 (*BCL2L1*, also called Bcl-xL), CASP8 and FADD like apoptosis regulator (*CFLAR*), caspase 3 (*CASP3*), Fas cell surface death receptor (*FAS*)*,* and actin beta (*ACTB*) by real-time RT-PCR (qPCR) using a SensiFAST SYBR Hi-ROX Kit (Bioline) on ABI Prism 7300 real-time PCR (Applied Biosystems, Thermo Fisher Scientific, Waltham, MA, USA). Gene expression levels normalized to *ACTB* as endogenous controls were calculated using the 2^−ΔΔCt^ method and expressed as mean ± SEM, relative to the unstimulated condition (control). The primer information used in the current study is listed in detail in Supplementary Table S1.

### 2.14. Western Blot

Total protein from control cell pellets as well as quercetin- and curcumin-treated milk PMNs was extracted with RIPA lysis buffer (Sigma-Aldrich, St. Louis, MO, USA) supplemented with protease inhibitor cocktail (Sigma-Aldrich, St. Louis, MO, USA). Protein concentrations were measured using a Bradford protein assay (Bio-Rad, Hercules, CA, USA). Protein samples were combined with 2× Laemmli Sample Buffer containing β-Mercaptoethanol (Bio-Rad, Hercules, CA, USA) and heated at 95 °C for 5 min. Equal amounts (30 μg) of protein were separated by 12% SDS-PAGE with Tricolor Broad Range Prestained Protein Ladder (Vivantis Technologies, Selangor Darul Ehsan, Malaysia). The gels were transferred to a 0.2 μm pore-size Immun-Blot PVDF Membrane (Bio-Rad, Hercules, CA, USA), followed by blocking in 5% bovine serum albumin (BSA, Bio Basic, Markham, ON, Canada) for 1 h. The membranes were incubated with purified mouse anti-caspase 3 [clone 4-1-18] monoclonal antibody (BioLegend, San Diego, CA, USA) for 2 h at RT and with HRP conjugated goat anti-mouse IgG (minimal x-reactivity) antibody (clone Poly4053, BioLegend, San Diego, CA, USA) for 45 min at RT. For signal detection, the PVDF membranes were developed using DAB detection. Bands were quantified with Image J software (NIH). Band optical density ratios were calculated relative to β-actin (Direct-Blot™ HRP anti-β-actin Antibody, BioLegend, San Diego, CA, USA) as a loading control.

### 2.15. Identification of Major Pathogens in Collected Bovine Milk by PCR

The aliquot of milk samples (1 mL) taken from the milk PMN isolation process were placed in a microcentrifuge tube and then centrifuged at 12,000 rpm, 3 min, at 4 °C (Hettich—Universal 320R, Kirchlengern, Germany). The liquid (whey) and semisolid portion (cream) were discarded. The cell pellet was resuspended in sterile PBS. Ten microliters of cell-pellet suspension were mixed with modified PEG–NaOH for bacterial genomic DNA (gDNA) extraction, according to a previously published protocol [17]. Two microliters (2 μL) of extracted gDNA were used as a DNA template for PCR amplification. PCR was performed in a C1000 Touch Thermal Cycler (Bio-Rad, Hercules, CA, USA). All reactions were carried out in a final volume of 25 μL composed of 6.25 μL of 2× MyTaq Hot Start Red Mix (Meridian Bioscience, Cincinnati, OH, USA), 1 μL of 10 μM of forward/reverse primer, and 14.75 μL H_2_O. PCR-cycling conditions were 1 cycle at 95 °C for 1 min, 30 cycles at 95 °C for 15 s, varying annealing temperature for 15 s, and 72 °C for 15 s, followed by 1 cycle of final extension at 72 °C for 2 min. A negative control containing all of the components of the reaction mixture without the DNA sample and a positive control containing 2 µL of *Staphylococcus aureus*, *Streptococcus agalactiae*, *Streptococcus uberis*, *Escherichia coli, Staphylococcus aureus* Coagulase-negative (CNS), and *Candida albicans* DNA were extracted by modified PEG–NaOH. Ten microliters of the PCR-amplified products were analyzed by electrophoresis on a 1% agarose gel in 0.5× TAE buffer stained with 0.5 μg of ethidium bromide/mL. Gel bands were documented using the GelMax Imager. The sequences of the oligonucleotide primers and PCR conditions used in this study [18,19,20,21,22] are listed in Supplementary Table S2.

### 2.16. Principal Component Analysis (PCA)

Principal component analysis (PCA) was applied to compare its usefulness with cluster analysis for evaluating the results obtained using cellular effector functions, as previously mentioned in detail, and gene expression profiles were analyzed by the real-time PCR of isolated milk PMNs treated with PBS, quercetin, and curcumin [23]. The PCA was the most suitable method that allowed for the reduction of the multidimensionality of the data, grouped the samples into three clusters, and made a possible selection of the most potent substances to be further used in bovine mastitis against *S. agalactiae* infection. Multivariate analyses and PCA were performed and generated by RStudio version 1.1.456, using the packages *ggfortify* and *cluster*.

### 2.17. Data Analysis

All experiments were performed 2 or 3 times and/or performed in triplicate. The Shapiro–Wilk normality test was used to determine the normality of the data by a Gaussian distribution. Most assays were evaluated using one-way ANOVA or the Kruskal–Wallis test followed by Tukey post hoc to compare treated groups (quercetin and curcumin) to control (PBS). GraphPad Prism 7.0 was used for all statistical analyses (GraphPad software, San Diego, CA, USA). Statistical significance was accepted where *p* < 0.05. Data presentations were displayed as mean with standard error (mean ± SE). Heat maps of the average gene expression levels were generated by R version 3.5.3, using the packages *gplots*, *viridis*, and *RColorBrewer*. Gene network was constructed using a web-based application (https://genemania.org/ accessed on 9 December 2020) that uses gene association to prioritize resources from curated and experimentally determined data. The GeneMANIA network was created using default settings with an automatically selected weighting method and based on the organism *Homo sapiens* (human). The protein–protein interaction network (PPI) was constructed using the STRING functional protein association networks (https://string-db.org/ accessed on 9 December 2020) with *Bos taurus* (domestic cow) protein reference database.

## 3. Results

### 3.1. Milk-Isolated Neutrophils and Identification of Bovine Mastitis-Causing Pathogenic Bacteria

**Figure 1:**
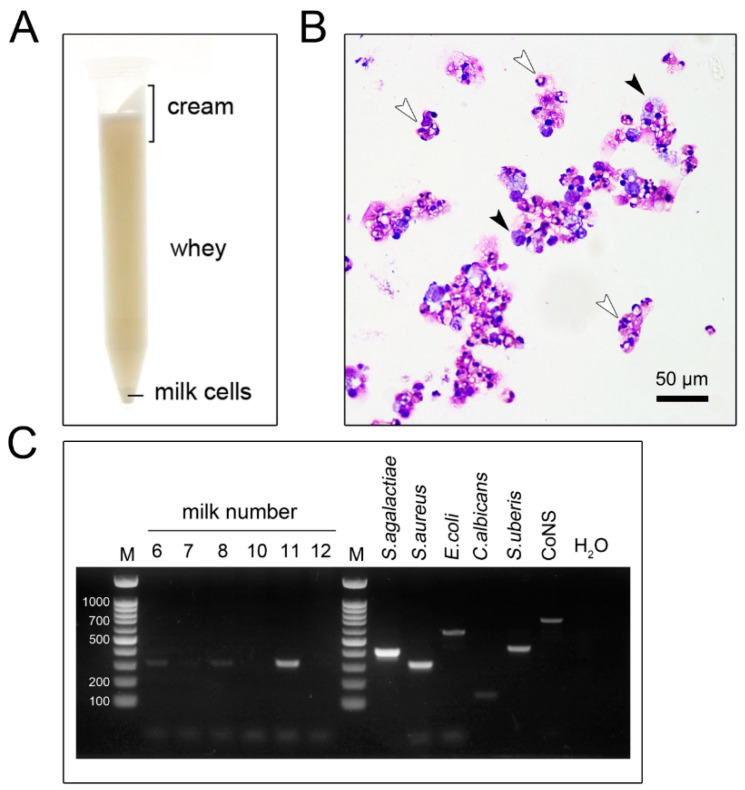
Bovine milk PMNs isolation and detection of bacterial genomic DNA in milk by conventional PCR. (**A**) Visualization of the separate fresh bovine milk layers using benchtop centrifuge. Milk cells were found at the bottom of the tube. (**B**) Cell morphology was revealed with cytospin slide preparation of milk PMNs stained with a Dip Quick Stain Set. The leukocytes were abundant in milk cells, and the majority of these cells were PMNs (white arrowheads) while heterogeneous cells that comprised macrophages (black arrowheads) were also visualized. The slides were viewed under a light microscope at magnification ×20. (**C**) Representative examples of PCR products from milk cells revealed the presence of *Staphylococcus aureus* genetic materials (16S ribosomal RNA gene; 16S rRNA) in some samples as described in Materials and Methods. Negative (H_2_O) and positive controls (16S rRNA of each bacterial species) were included in each PCR reaction. Coagulase-negative staphylococci (CoNS).

We successfully isolated milk-isolated neutrophils in accompanying macrophages from quarter-milk samples that tested positive via a California mastitis test (CMT)( Figure 1A–C). The purity of isolated milk cell pellets was later identified by cytospin slide preparation, as depicted in Figure 1B and by flow cytometry. The average numbers of total isolated cells per milliliter of raw milk were ranked from 5 to 19 × 10^6^ cells per mL. To reliably identify any particular contaminating bacteria species and yeast in our milk samples, we used PCR to amplify genus-specific ribosomal RNA. The presence of known bacteria in the milk samples was also obtained from the milk cultures. The PCR results indicated that many of the milk samples contained *Staphylococcus aureus*, whereas most milk samples were free from pathogenic bacteria and yeast (Figure 1C). The PCR result followed the results found in the bacterial and yeast cultures (Figure 1C). To this end, the milk samples in this study were determined free of *Streptococcus agalactiae* that may have caused some interference of cells in subsequent experiments.

### 3.2. Streptococcus agalactiae Viability after Being Treated with Different Concentrations of Quercetin/Curcumin

**Figure 2:**
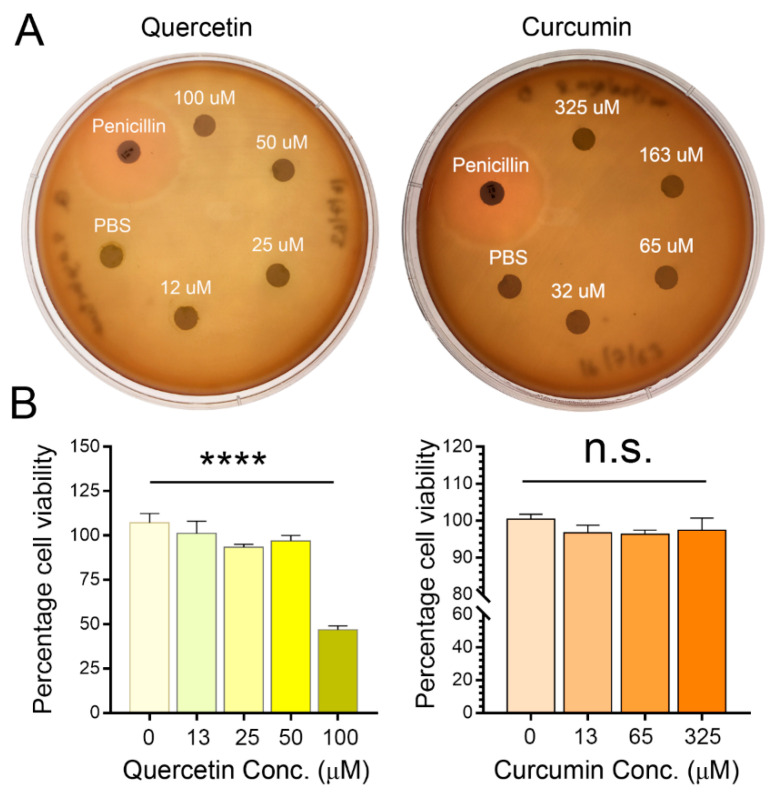
Tests of the direct effects of quercetin and curcumin antibacterial activity on *Streptococcus agalactiae* by disk diffusion assay and the MTT cell viability assay of milk PMNs after incubation with test compounds. (**A**) Antibacterial activity by disk diffusion assay using 6 mm filter paper disks dipped in PBS, known concentrations of either quercetin (12, 25, 50, and 100 μM) or curcumin (32, 65, 163, and 325 μM), and penicillin G on Mueller Hinton Agar (MHA). (**B**) Cell viability was assessed by MTT assay. No toxic effects on cell viability up to 50 μM could be seen for all quercetin and curcumin concentrations. All experiments were performed twice. Data expressed as the mean ± SEM (*n* = 8 each treatment) (Table S3), one-way ANOVA, **** *p <* 0.0001, n.s., not significant.

The experimental results demonstrated that the direct bactericidal efficiency of quercetin/curcumin on *Streptococcus agalactiae* was non-existent at any concentration via the agar diffusion method, as compared to antibiotics (Figure 2A). In our judgment, the bacteria were insensitive to direct killing regardless of the quercetin/curcumin concentrations.

### 3.3. Quercetin/Curcumin Showed No Cytotoxic Effects on Milk-Isolated Neutrophils

To investigate whether quercetin/curcumin had any direct toxicity on cell viability, we supplemented varying doses of either quercetin (0–100 μM) or curcumin (0–325 μM) and then performed MTT assays (Figure 2B). The results demonstrated that quercetin had no role in promoting cell death at a concentration lower than 100 μM (Figure 2B). The percentage of cell viability was more than 93.42 in 0–50 μM of quercetin, whereas a cell viability of 47% was reported at 100 μM of quercetin. In contrast, curcumin had no influences on cell viability at every tested concentration (Figure 2B). The percentage of cell viability after being treated with curcumin was more than 96.3. We have already predetermined the quercetin concentration at 50 μM, according to our previous study of bovine neutrophils. A suitable curcumin concentration to be used in milk PMNs has not been previously determined. In selecting the optimal curcumin concentration for promoting neutrophil effector functions, we had conducted a preliminary examination. It was predefined that 65 μM of curcumin was highly amenable to promoting cellular function. In the subsequent experiments, a 50 μM final concentration of quercetin and a 65 μM final concentration of curcumin were used.

### 3.4. Quercetin/Curcumin Increased Cell Motility toward Streptococcus agalactiae

**Figure 3:**
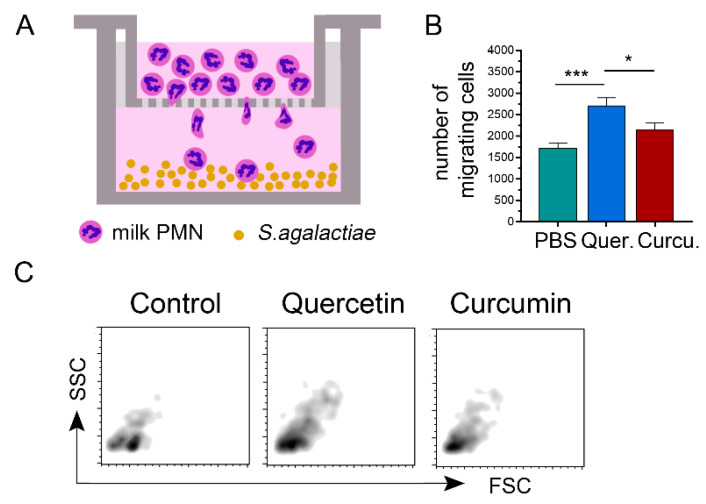
In vitro Transwell migration assay of milk polymorphonuclear neutrophil leukocytes (PMNs) treated with quercetin or curcumin toward *Streptococcus agalactiae*. (**A**) A schematic diagram depicting procedures of milk PMNs migration in a Transwell setup. (**B**) The flow cytometer forward (FSC) versus side scatter (SSC) plots of migrating cells in PBS, served as control, quercetin-treated, and curcumin-treated cells. (**C**) Histogram comparing the mean number of migrating cells in each treatment group from two independent experiments. Data expressed as the mean ± SEM (*n* = 15 each treatment), one-way ANOVA followed by Tukey’s multiple comparisons tests, * *p <* 0.05, *** *p <* 0.001.

We next assessed the dynamic cell motility toward live *Streptococcus agalactiae* of the quercetin-treated/curcumin-treated milk PMNs in the Transwell experiments (Figure 3A‒C). The numbers of migrated cells with PBS, quercetin-treated, and curcumin-treated were 1717 ± 122.4, 2708 ± 191.7, and 2155 ± 155.3, respectively (Figure 3B). Overall, the transmigration of the treated cells presented in this report significantly differed from the PBS control (*p =* 0.0003, Figure 3B).

### 3.5. Quercetin/Curcumin Mitigated the Level of Intracellular Reactive Oxygen Species (ROS) of Milk-Isolated Neutrophils

**Figure 4:**
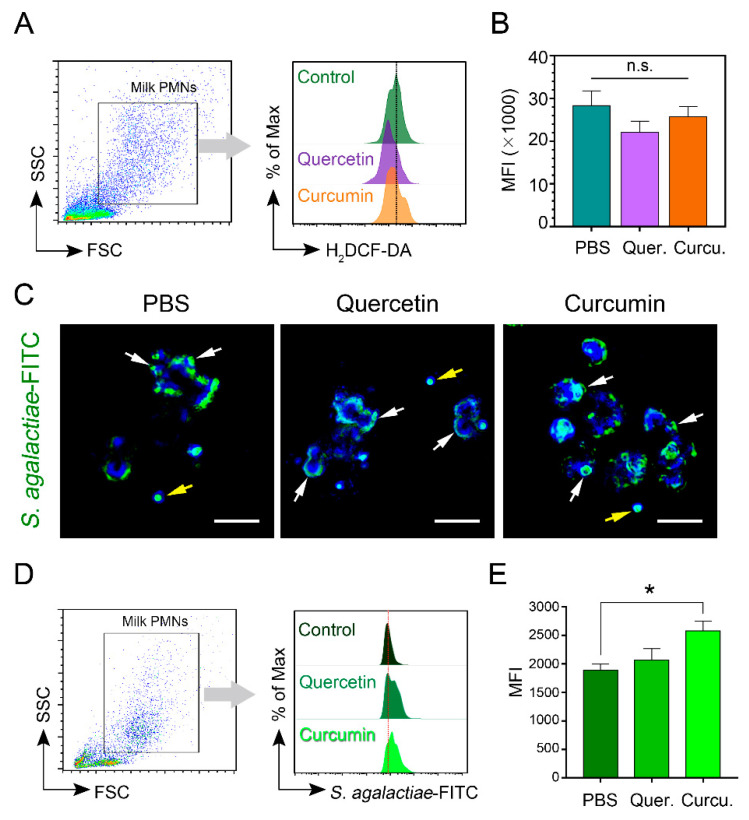
Reactive oxygen species (ROS) generation and phagocytosis of *S. agalactiae* in bovine milk PMNs. Cells were treated with either 50 μM quercetin or 65 μM curcumin, and the measurements were mainly performed by flow cytometer, as indicated in the Section 2. (**A**) Gating of milk PMNs and representative half-offset histograms (on the right) showed mean fluorescence intensity (MFI) of ROS-positive cells from each treatment. (**B**) Bar graphs represent the mean MFI of ROS-positive cells. (**C**) Representative fluorescent images of phagocytosis of milk PMNs co-cultured with FITC- *S. agalactiae* in PBS, quercetin, and curcumin treatments. White arrows indicate the cells with internalized bacteria. *Yellow arrows* indicate extracellular bacteria. ×20 magnification, scale bars = 20 μm. (**D**) Gating of milk PMNs and representative offset histograms show MFI of phagocytosis-positive cells from each treatment. (**E**) Bar graphs represent the mean MFI of phagocytosis-positive cells. Results are inclusive of two separate experiments. Data in (**B**,**E**) presented as mean ± SEM (*n* = 18–19 each treatment), One-way ANOVA followed by Tukey’s multiple comparisons test, * *p <* 0.05, n.s., not significant.

To assess the effects of quercetin/curcumin on the generation of intracellular reactive oxygen species (ROS), we measured primarily intracellular hydrogen peroxide (H_2_O_2_) with fluorescent dye (H_2_DCF-DA), which was analyzed by flow cytometry (Figure 4A). Flow cytometry data (mean fluorescence intensity, MFI) showed that the cells treated with 50 μM quercetin reduced the amount of ROS production (MFI of 22,060 ± 2620), as compared to the PBS control cells (MFI of 28,320 ± 3480; *p <* 0.304, Figure 4B). In addition, the results presented here showed that the ROS level was also lower in the 65 μM curcumin sample (MFI of 25,690 ± 2380), as compared to the PBS (Figure 4B).

### 3.6. The Process of Internalization and Phagocytosis of S. agalactiae by Milk PMNs Was Increased by the Action of Quercetin or Curcumin

The phagocytosis of FITC-labeled *S. agalactiae* by milk PMNs supplemented with either quercetin or curcumin was studied in vitro. As with the phagocytosis of pathogenic bacteria by circulating bovine neutrophils, the phagocytosis of bacteria by milk PMNs was comparable to the phagocytosis of their neutrophil counterparts. The milk PMNs appeared to be more robust in curcumin-treated cells, as compared to quercetin-treated cells as well as control cells (Figure 4C,D). Encounters with bacteria resulted in significantly enhanced phagocytosis in the stimulating cells (MFI of 2069 ± 199.4 for quercetin and MFI of 2581 ± 170.3 for curcumin) versus the PBS controls (MFI of 1897 ± 100.7; *p <* 0.012, Figure 4D). Fluorescent images confirmed phagocytosis of opsonized fluorescently labeled *S. agalactiae* in the treated cells versus the controls (Figure 4E).

### 3.7. In Vitro Treatment of Milk PMNs with Either Quercetin or Curcumin Enhanced Bacterial Killing

**Figure 5:**
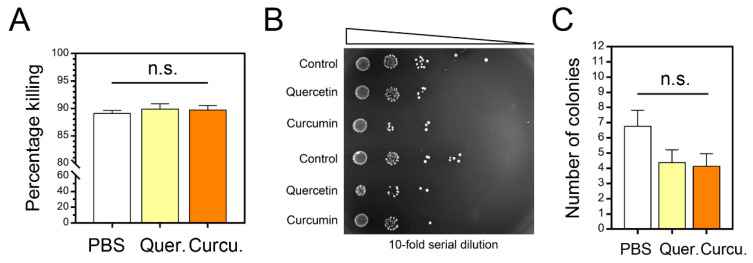
Bactericidal activity of bovine milk PMNs treated with PBS, quercetin, and curcumin. (**A**) The bar graphs show the results obtained in the MTT assay in cells treated with each test compound. (**B**) Spot dilution assay determined the viability of *S. agalactiae* after uptake and killing by milk PMNs. Sample were aliquots from the same samples used for MTT assay after milk PMN cell lysis, as indicated and detailed in Section 2. Liquid samples were spotted at 10-fold serial dilutions (indicated by triangles). (**C**) Numbers of viable bacteria were enumerated by colony counting on agar plates from the third serial dilution (10^−3^) and presented by bar graphs. Results were inclusive of two separate experiments. Data in (**A**,**C**) presented as mean ± SEM (*n* = 16–17 each treatment), one-way ANOVA, n.s., not significant.

For the extracellular bacterial killing of milk PMNs, we performed an MTT assay to measure the bacterial viability as the assessment of the capability of quercetin- and curcumin-treated cells to annihilate the harmful bacteria. However, as shown in Figure 5A, the MTT assay revealed that the milk PMNs innately killed live *S. agalactiae* that had not been treated or supplemented. The in vitro supplementation of either quercetin or curcumin appeared not to be significantly effective in this test model to facilitate the killing of encountered bacteria. Treated cells did not significantly destroy surrounding bacteria among treatments (*p =* 0.768, Figure 5A).

The killing of bacteria was also confirmed by examining the bacterial colony spotted onto nutrient agar plates in serial spot-dilution assays. The results demonstrated neutral sizes of bacterial colonies among the control (PBS), quercetin-treated, and curcumin-treated cells (Figure 5B). At 10^0^ and 10^1^ dilutions, samples were recorded as too numerous to count (TNTC) in all treatments. We found that 10^2^ dilutions exhibited the countable number of colonies in the range of 3–11 per spot in PBS and 1–8 in quercetin and curcumin. There was no statistically significant difference among these countable colonies in different treatment groups (*p =* 0.108, Figure 5C). Overall, our data showed no clear bacterial-killing capacity of the milk PMNs after treatments.

### 3.8. The Formation of NETs by Milk PMNs Was Triggered by Quercetin/Curcumin Supplementation

**Figure 6:**
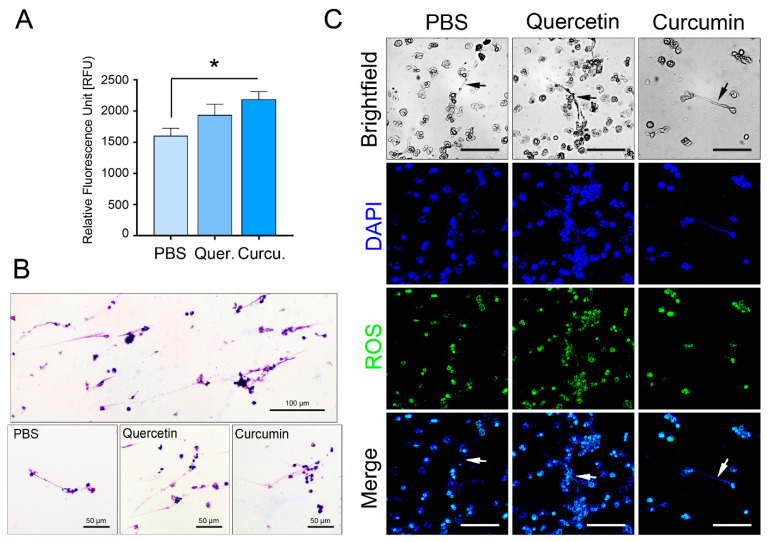
Neutrophil extracellular traps (NETs) in milk PMNs treated with quercetin and curcumin. NETs were detected using a fluorescence plate reader, light, or fluorescence microscope. (**A**) Relative fluorescence unit (RFU) and quantitative comparison of the amounts of NET release by quercetin-, curcumin-treated cells compared with PBS. The amounts of NETs were significantly higher in quercetin and curcumin groups. (**B**) Representative images of fibrous structures produced extracellularly from activated milk PMNs (NETs) in the presence of PBS, quercetin, and curcumin and later activated with live *S. agalactiae*. ×20 magnification. (**C**) Representative images of NETs captured by fluorescent microscopy after staining the cells with DAPI for DNA, and H_2_DCF-DA for ROS. Cells were incubated with quercetin, curcumin, and PBS. Arrows indicated NETs, ×20 magnification, scale bars = 50 μm. Data in (**A**) expressed as the mean ± SEM (*n* = 19 each treatment), one-way ANOVA followed by Tukey’s multiple comparisons tests, * *p <* 0.05.

The presence of extracellular pathogens may stimulate the formation of fibrous structures composed of genetic materials, granule enzymes, and harmful constituents inside PMNs. The indirect killing of extracellular pathogens by NETs was examined after the cells were supplemented with PBS, quercetin, or curcumin by a fluorescence plate reader. Both the quercetin-primed (1935 ± 177.9 RFU) and curcumin-primed cells (2185 ± 129.6 RFU) released significant numbers of NETs, as compared to the unstimulated cells (1597 ± 127.1 RFU, *p =* 0.023, Figure 6A).

The extracellular structures were also visually confirmed by a fluorescent microscope (Figure 6B,C). We noticed that the long, stretched NETs tended to project away from the center of the cell regardless of the substance used. The ROS molecules were also scarcely detected in NET generated by quercetin-stimulated cells, as depicted in Figure 6B, middle panel.

### 3.9. Patterns of Gene Expression in Milk PMNs Stimulated with S. agalactiae Were Altered by Supplementation of Quercetin/Curcumin

**Figure 7:**
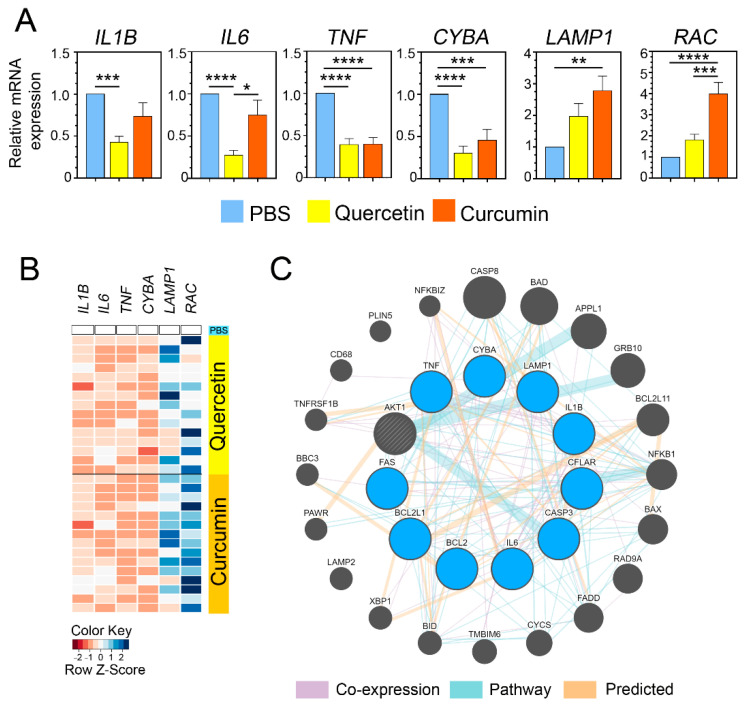
Gene expression analysis of proinflammatory cytokine genes and genes involved in effector functions. Quantitative real-time PCR analyses indicated gene expression changes in cells that received either quercetin or curcumin, as compared to PBS. (**A**) Proinflammatory cytokine genes *IL1B*, *IL6*, *TNF*, and *CYBA* for ROS subunit gene were significantly down-regulated in cells treated with either quercetin or curcumin, whereas a gene of either lysosomal marker (*LAMP1*) or migration-related gene (*RAC*) was up-regulated, which correlated with a significant increase of phagocytosis and cell migration. (**B**) Heatmap of gene expression across a panel of PBS, quercetin-, and curcumin-treated cells corresponding to all observed data from (**A**). Expression level was scaled, as indicated, by row z-score with dark blue indicating increased expression and brick orange indicating decreased expression. (**C**) Gene association network by GeneMANIA for 10 up- or down-regulated genes. Input genes (deep sky blue) were analyzed using the default setting. Relationship among input genes and predicted genes by GeneMANIA (small and large dark grey circles) were connected by line colors according to the type of interaction (i.e., co-expression, pathway, predicted), as explained in the legend on the bottom of the figure. Data in (**A**) presented as mean ± SEM (*n* = 14–15 each treatment), one-way ANOVA followed by Tukey’s multiple comparisons test, * *p <* 0.05, ** *p <* 0.01, *** *p <* 0.001, **** *p <* 0.0001.

We monitored the alteration of gene expression in the milk PMNs with and without the supplementation of either quercetin or curcumin. The genes involved in proinflammation (e.g., *IL1B*, *IL6*, *TNF*), ROS, and phagocytosis (e.g., *CYBA*, *LAMP1*, *RAC*) were analyzed for the levels of expression among the treatments. Our findings indicated that the expressions of all three tested genes involved in proinflammation were significantly down-regulated in the quercetin-treated as well as the curcumin-treated milk PMNs (Figure 7A, *IL1B*, *IL6*, *TNF*). Specifically, all the genes in the cells treated with quercetin were decreased by over 50%. The expression of *IL1B*, *IL6*, and *TNF* in the quercetin-treated cells was suppressed by 58%, 73%, and 61%, respectively. Similarly, the expressions of the aforementioned genes in curcumin treatments were mildly decreased. The fold suppression of the genes *IL1B*, *IL6*, and *TNF* in the curcumin group was 26%, 25%, and 50%, respectively. In addition, the *CYBA* gene that participated in the ROS generation was prevented by the action of quercetin (0.302-fold) and curcumin (0.455-fold) in the milk PMNs (Figure 7A). In contrast, a significant elevation of the genes involved in phagocytosis (i.e., *LAMP1* and *RAC*) to clear bacteria was observed in both the quercetin and curcumin groups (Figure 7A). The genes involved in phagocytosis were elevated between 1.965-fold and 2.778-fold (Figure 7A) for *LAMP1*, and between 1.810-fold and 3.997-fold in the treatment groups (Figure 7A) for *RAC.* To review the expression patterns described above, a heat map was generated using the qPCR data, and it depicted a z-score scale of relative mRNA abundance after the exposure of the cells to either quercetin or curcumin across all the samples, according to a color scale (Figure 7B). GeneMANIA showed a circular network and a subnetwork based on our query list (*IL1B*, *IL6*, *TNF*, *CYBA*, *LAMP1*, *RAC, CASP3, FAS, CFLAR, BCL2,* and *BCL2L1*) and the predicted genes, the co-expressed genes, and the gene sets in the sample pathway (Figure 7C). The network output of known and predicted relationships showed that our query genes were related to the top 20 genes, all of which had apoptosis-related functions, mitochondrial membrane permeability, and a cellular response to mechanical stimulus.

### 3.10. Quercetin/Curcumin Manipulated Milk PMN Cell Death

**Figure 8:**
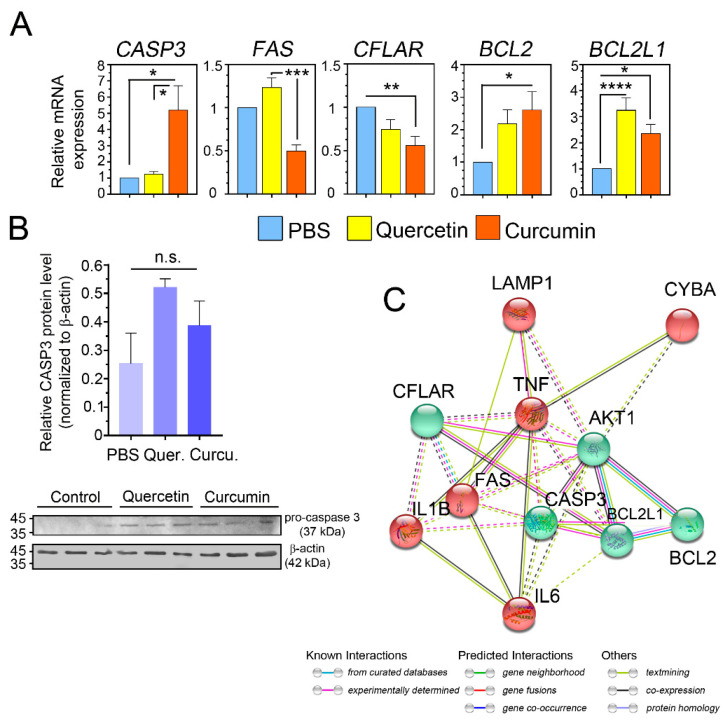
Quercetin and curcumin may affect programmed cell death in isolated milk PMNs. (**A**) Differentially expressed genes (DEGs) related to cell death (both proapoptotic and antiapoptotic genes) in milk PMNs treated with quercetin and curcumin were analyzed by quantitative real-time PCR. Gene expression of the treated milk PMNs during *S. agalactiae* infection revealed the modulation of the expression of selected proapoptotic genes (*CASP3*, *FAS*, and *CFLAR*) and antiapoptotic genes (*BCL2* and *BCL2L1*). (**B**) Effect of quercetin and curcumin on CASP3 protein expression by Western blots. ((**B**), *upper panel*) The CASP3 protein expression levels were detected by WB. Two herbal compounds induced the increased CASP3 activity versus PBS control. ((**B**), *lower panel*) Representative WB performed with the anti-CASP3 and anti-β actin antibodies (two independent experiments, *n* = 3 each treatment). (**C**) The STRING protein-protein interaction network of 10 up- or down-regulated genes in quercetin- or curcumin-treated cells. STRING network representing the predicted functional partners of the query proteins. Apoptotic proteins dominated by CASP3, BCL2, BCL2L1, CFLAR, and AKT1 were found to have interacted in one cluster (green). Proteins whose functions in proinflammatory cytokine, phagocytosis, and ROS generation (IL1B, IL6, TNF, CYBA, LAMP1, and FAS) clustered into another group (red). Different types of interactions are depicted by different colored lines. The legend of the interaction network is summarized in the figure. These proteins interacted with each other as well as with some other predicted functional proteins. Data in (**A**) presented as mean ± SEM (*n* = 15 each treatment), one-way ANOVA followed by Tukey’s multiple comparisons test, * *p <* 0.05; ** *p <* 0.01; *** *p <* 0.001; **** *p <* 0.0001; n.s., not significant.

We then investigated the fate of the cells after treatment with the test compounds and subsequently challenged with bacteria. Alterations in gene expression were determined by the balance between proapoptotic (*CASP3*, *FAS*, *CFLAR*) and antiapoptotic genes (*BCL2*, *BCL2L1,* also known as Bcl-xL) in the current study. We evaluated a set of three genes involved in the intrinsic pathway of apoptosis, namely *CASP3* and *FAS* as the death receptor as well as the CASP8 and FADD-like apoptosis regulator (*CFLAR*). Notably, the expressions for two out of the three pro-apoptotic genes (*CASP3*, *FAS*) were significantly upregulated (*p =* 0.0065 and *p* < 0.0001, respectively, Figure 8A), whereas the expression of *CFLAR* was significantly reduced in the cells treated with the test compounds (*p =* 0.004, Figure 8A). The survival of the milk PMNs exposed to the test compounds and bacteria were determined in part by real-time PCRs. As members of the Bcl-2 family, *BCL2* and *BCL2L1* (Bcl-xL) acting as anti-apoptotic genes were found in cells treated with quercetin and curcumin. The induction of these two anti-apoptotic genes was observed in either quercetin- or curcumin-treated cells (Figure 8A). A significant increase in the expression of *BCL2* by more than 2-fold (2.176-fold) in quercetin-treated cells and 2.60-fold in curcumin-treated cells was revealed (*p =* 0.0247, Figure 8A). Similarly, the *BCL2L1* gene had also significantly changed more than two-fold in treated cells (*p =* 0.0001), as compared to the controls (Figure 8A).

In this report, we examined if either quercetin or curcumin treatments of cells with *S. agalactiae* infection would lead to the induction of cell death, which occurs, in part, via apoptosis. The levels of procaspase 3 (CASP3) protein expression were analyzed based on protein lysates by Western blot analysis using an antibody capable of detecting either procaspase 3 or cleaved caspase 3. The conversion of procaspase 3 to active (i.e., cleaved) caspase 3 was also assessed using Western blot analysis. Furthermore, the band intensity was normalized with β-actin. The results showed that the two test compounds could potentially increase the proapoptotic proteins (Figure 8B). The levels of procaspase 3 protein expression in the milk PMNs treated with the test compounds were 0.52-fold (quercetin) and 0.38-fold (curcumin), as compared to the PBS (0.25-fold) control cells ( *p <* 0.101, Figure 8B). The levels of cleaved caspase 3, which would reflect the degree of apoptosis of cells, were not detected in any cell treatments. The protein bands at 17 kDa, which corresponded to cleaved caspase 3, were not observed.

We sought to identify the protein networks and the pathways that may act in concert and in association with milk PMNs’ innate functions. The protein network was constructed and visualized using STRING network-based tools. STRING uses protein names to search for known and predicted protein interactions. The input set of protein names containing IL1B, IL6, TNF, CYBA, LAMP1, RAC, CASP3, FAS, CFLAR, BCL2, and BCL2L1 was queried and filtered. The intermediate and final results were retrieved from the database. We utilized the existing databases of protein networks and pathways to gain insight into the processes related to the modulatory effects of the test compounds. The analyzed protein networks dictated the functional associations of the proteins from inflammatory responses, phagocytosis, ROS biosynthesis, and apoptosis (Figure 8C).

The gene ontology (GO) terms corresponding to the genes with a known function were derived by STRING analysis. The top GO biological process (BP) categories were necroptotic signaling pathway (GO:0097527), positive regulation of acute inflammatory response (GO:0002675), positive regulation of phagocytosis (GO:0050766), and positive regulation of ROS biosynthesis (GO:1903428). The top molecular function (MF) categories were cytokine activity (GO:0005125), cytokine receptor binding (GO:0005126), and protein heterodimerization activity (GO:0046982). Cellular component (CC) categories were autophagosome (GO:0005776), external side of the plasma membrane (GO:0009897), and plasma membrane protein complex (GO:0098797). All interactions were considered significant with a false discovery rate (FDR) of less than 0.01. When clustering was applied to the protein networks using the *k*-mean clustering method, two distinct clusters were generated, which were composed of the proteins IL1B, IL6, TNF, FAS, CYBA, and LAMP1 in one cluster (red circles) and BCL2, BCL2L1, CASP3, CFLAR, and AKT1 in the other cluster (green circles), (Figure 8C).

### 3.11. Principal Component Analysis (PCA) Showed a Clear Separation among Control, Quercetin-Treated, and Curcumin-Treated Milk PMNs Based on Effector Functions and Gene Expressions

**Figure 9:**
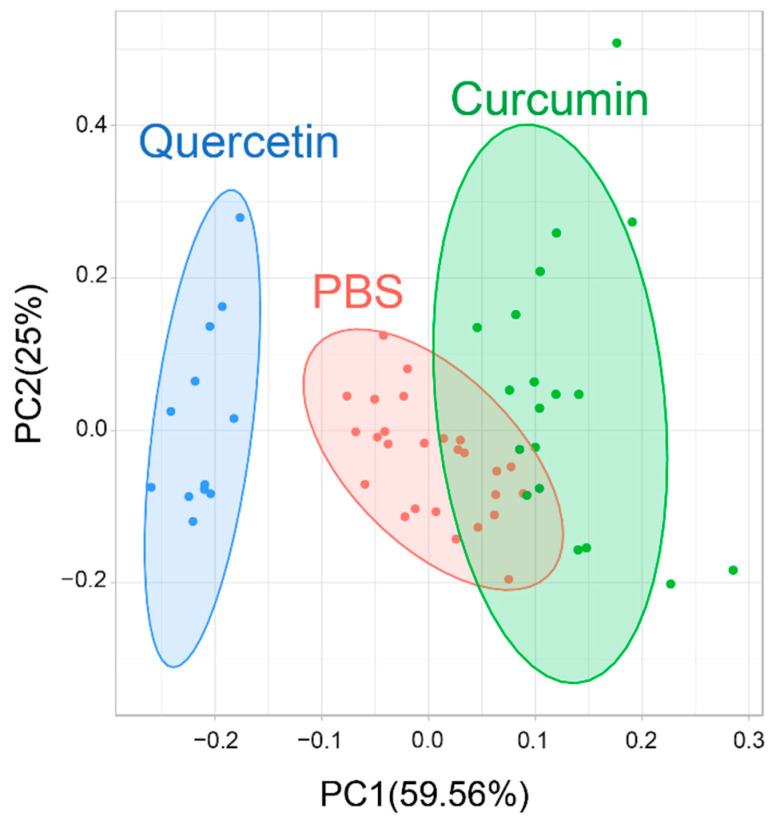
Principal component analysis (PCA) for feature selection based on 14 cellular and molecular activities. PCA compared the influences of quercetin versus curcumin on milk PMNs, and versus control (PBS).

As the results from the quercetin-treated and curcumin-treated milk PMNs showed that their effector functions and a group of genes’ expression were altered, a multivariate analysis was conducted to identify any superior benefits that could be attributed to these test compounds. We performed an unsupervised principal component analysis (PCA) for all three datasets of cellular functions and the gene expression data from the unstimulated control, quercetin-treated, and curcumin-treated milk PMNs. The PCA graphical interpretation showed that only the first two principal components (PCs) were used for further consideration (Figure 9). Comparing the parameters obtained for the three datasets in question, it was observed that the first two PCs together explained the highest variance (approximately 84%) in the three datasets. While evaluating the results obtained regarding the efficacy of the supplementation of quercetin and curcumin, we considered whether the data was justified according to the maximum variance criterion.

The three groups were indicated by separate ellipses. The proportion of variance captured was given as a percentage for both the first (PC1) and second component (PC2). The first two PCs accounted for more than 80% of the variance.

To identify the potential use of the two studied substances, the PCA data showed a clear separation among the three datasets by PC1, and the delineation of all three datasets was identified. The quercetin-treated cells were the only group that demonstrated a clear separation from the other two groups. These results indicated that quercetin may be suitable for supplementation with the intent of increasing both cellular and molecular functions to combat bacterial mastitis. The interpretation of the experimental data obtained in this study confirmed the use of quercetin in bovine mastitis.

## 4. Discussion

Several plant compounds such as resveratrol, quercetin, and curcumin have been shown to have potential as immunomodulators [5,6]. The extracts obtained from many fruits, vegetables, leaves, and grains contain quercetin and curcumin and have been used in traditional medicine to boost immunity. In addition, quercetin was previously shown to enhance innate immunity in bovine neutrophils [3,4,13]. The current study examined whether these two compounds had the potential to improve innate immune function during the course of bovine mastitis, but this was not fully documented in our in vitro study. Our cumulative results indicated that quercetin and curcumin might enhance overall milk PMN functions that had been foretold by respective composite data. The study uncovered differences in milk PMN functional patterns among the control, quercetin-, and curcumin-treated cells, particularly cell migration, phagocytosis/bacterial killing, and NET production. We observed that ROS scavenging in the treated cells was slightly diminished via intracellular detection and down-regulation of the ROS-subunit gene. This finding agreed with previous reports in mice and cows; however, our finding regarding ROS scavenging did not have a sufficient suppressive effect [3,24]. Both quercetin and curcumin appeared to reduce ROS formation in neutrophils associated with oxidative stress and inhibit mitochondrial permeability transition as an early event in apoptosis. However, further studies are needed to define the role of ROS inhibition at the protein level. We further assessed the role of the tested compounds in the forward motion of the cell. As was consistent with our previous report as well as another [15], we demonstrated that quercetin potentially facilitates immune cell migration toward fungal pathogens. A recent report by Madhyastha et al. also found that quercetin promoted wound healing in a fibroblast model [25]. In contrast to other reports in cancer cells, the reports suggested that, under most circumstances, these natural products significantly attenuated cell migration and invasion, perhaps via the Rho GTPases Rac and Cdc42 [7,26,27,28]. Gene expression data also showed changes in a subset of genes (*CYBA*, *LAMP1*, *RAC*) that are involved and required for oxidative burst and phagocytosis killing. Therefore, the cytoskeletal regulation by RAC may have contributed to the enhanced cell migration, phagosome formation, efficient killing of internalized bacteria, and production of NET, which were correlated with the findings from many effector-function assays. The main limitation in our experiment was that we could not perform such experimentation inside living cows. Further studies should attempt to establish whether this positive effect also occurs in vivo. For the gene expression aspects, quercetin and/or curcumin mitigate proinflammatory cytokines *IL1B*, *IL6*, *TNF*, and the gene involved in ROS, *CYBA* [5,8,13,15,29]. These results were consistent with previous outcomes regarding the cellular function of ROS generation [15]. For other functional genes, both compounds upregulated *LAMP1* and *RAC* genes, as were the findings in our previous data involving bovine neutrophils [3,4].

For controlling cell survival and apoptosis, the detection of caspase-mediated cell death led to the conclusion that upregulation genes *CASP3* and *FAS* (CD95) in quercetin- and/or curcumin-treated cells may somehow induce the mitochondrial-dependent caspase-3-apoptotic pathway [30,31]. Increased expression of specific proteins in the FAS/caspase-8 pathway has also been reported in human studies after curcumin treatment [31]. Our results illustrated the upregulation of the *FAS* gene only in quercetin treatment, whereas relative concentrations of procaspase 3 protein were increased in both treatments (Figure 8). The downregulation of *CFLAR*, as the inhibitor of TNFRSF6-mediated apoptosis, after treatment indicated the promotion of the FAS/caspase-8-apoptotic pathway [32]. The role of the Bcl-2 family proteins (Bcl-2 and Bcl-xL) in curcumin-induced apoptosis remains unclear [31,33]. The upregulation of *BCL2* and *BCL2L1* (Bcl-xL) in anti-apoptotic roles did not inhibit the induction of apoptosis in milk PMNs in the current study. Based on our observations, a more effective way to trigger programmed cell death via caspase activation in milk PMNs would be via the engagement of surface receptor FAS by FasL results in apoptotic cell death through FAS and caspase 8, procaspase 3, and cleaved caspase 3, and then entering full apoptosis. However, whether milk-derived neutrophils have a short lifespan, easily change cell fate, or undergo early cell death are discussion points to ponder.

## 5. Conclusions

We concluded, from a resource perspective, that quercetin could have potential in the development of a cure for bovine mastitis. As the purpose of the current study was to highlight the effects of the plant compounds quercetin and curcumin, we were not able to ascertain a direct association between their effects and changes in milk PMNs’ functions in vivo. To the best of our knowledge, neither quercetin nor curcumin, nor their derivatives, are currently being used for the treatment of bovine mastitis. However, further investigations, including in vivo studies, should be pursued to better understand the potential of quercetin and curcumin in the treatment of bovine mastitis.

## Data Availability

Not applicable.

## Supplementary Materials

The following are available online at https://www.mdpi.com/article/10.3390/ani11113286/s1. Table S1. Details of real-time PCR primer sequences; Table S2. Details of PCR primers for detection of 16S rRNA genes (bacteria) or 18S rRNA of the gene of yeast species; Table S3. All raw data.

## Abbreviations

PMN: polymorphonuclear neutrophil leukocytes; NETs: neutrophil extracellular traps; SCC: somatic cell count; CMT: California mastitis test; FBS: fetal bovine serum; MTT: 3-[4,5-dimethylthiazole-2-yl]-2,5-diphenyltetrazolium bromide; ROS: reactive oxygen species; HBSS: Hanks’ balanced salt solution; IL1B: interleukin 1 beta; IL6: interleukin 6; TNF: tumor necrosis factor; CYBA: cytochrome b-245 alpha chain; LAMP1: lysosomal associated membrane protein 1; RAC; Ras-related C3 botulinum toxin substrate; BCL2: B-cell CLL/lymphoma 2; BCL2L1: BCL2 like 1; CFLAR: CASP8 and FADD like apoptosis regulator; CASP3: caspase 3; FAS: Fas cell surface death receptor; FASL: Fas ligand; ACTB: actin beta; BSA: bovine serum albumin; PCA: principal component analysis; MFI: mean fluorescence intensity; RFU: relative fluorescence unit.
