# Supplementary material for: Immunomodulatory Effects of Herbal Compounds Quercetin and Curcumin on Cellular and Molecular Functions of Bovine-Milk-Isolated Neutrophils toward Streptococcus agalactiae Infection
Source: Animals (Basel). 2021 Nov 17;11(11):3286. doi: 10.3390/ani11113286 (PMC8614355; doi:10.3390/ani11113286)
Supplement: Supplementary file 1 [file animals-11-03286-s001.zip › animals-1435788 (3).pdf]

Supplemental Tables

Table S1. Details of real-time PCR primer sequences

Gene	Accession No.	Primer	Sequence	Size (bp)	Reference
IL1B	NM_174093	Forward	ACAAAAGCTTCAGGCAGGTG	226	[13]
		Reverse	AGCACCAGGGATTTTTGCTC		
IL6	NM_173923	Forward	AGCGCATGGTCGACAAAATC	179	[13]
		Reverse	AGCAGTGGTTCTGATCAAGC		
TNF	NM_173966	Forward	AGCACCAAAAGCATGATCCG	226	[13]
		Reverse	TTTGAACCAGAGGGCTGTTG		
CYBA	NM_174034	Forward	TCAGTTCACCCAGTGGTACC	135	[4]
		Reverse	ACTCTGGTCAGGTACTTCTGTC		
LAMP1	NM_001075124	Forward	ACAACGTTTCTGGCAGCAAC	125	[4]
		Reverse	GGTCTTGTTGGGGTTGACATTG		
RAC1	NM_174163	Forward	TGCCAATGTCATGGTGGATG	193	[4]
		Reverse	ACAATGGTGTCGCACTTCAG		
BCL2	NM_001166486	Forward	GTATGGCCCTAGCATGCGG	116	This study
		Reverse	ACTTATGGCCCAGATAGGCA		
BCL2L1	NM_001077486	Forward	TGTGGCCTTTTTCTCCTTCG	108	This study
		Reverse	TCATTCAGGTAAGTGGCCATCC		
CFLAR	NM_001012281	Forward	ACTCACTCTGGGGTCCCTTT	122	This study
		Reverse	AGCTGGCCCTCTGATTTCAC		
ACTB	NM_173979	Forward	TGCGGCATTCACGAAACTAC	146	[13]
		Reverse	AGGGCAGTGATCTCTTTCTGC		

Table S2. Details of PCR primers for detection of 16S rRNA genes (bacteria) or 18S rRNA of the gene of yeast species

Gene	Accession No.	Primer	Sequence	Size (bp)	Annealing Temp (°C)	Reference
rrsA *Escherichia coli* 16S rRNA[1]	NC_000913.3	Forward<br>Reverse 1<br>Reverse 2	GGGAGTAAAGTTAATACCTTTGCTC<br>TTCCCGAAGGCACATTCT<br>TTCCCGAAGGCACCAATC	584	56/60	[21]
Staphylococcus aureus 16S rRNA[2]	CP000255.1	Forward<br>Reverse	AGGATTAGATACCCTGGTAGTCCA<br>ACTTAACCCAACATCTCACGACAC	315	61	This study
Streptococcus agalactiae 16S rRNA[3]	NR_040821.1	Forward<br>Reverse	CGCTGAGGTTTGGTGTTTACA<br>CACTCCTACCAACGTTCTTC	405	58	[18]
Streptococcus uberis 16S rRNA[4]	U41048.1	Forward<br>Reverse	CGCATGACAATAGGGTACA<br>GCCTTTAACTTCAGACTTATCA	445	58	[19]
Coagulase-negative staphylococci (CoNS) 16S rRNA[5]	D83355.1	Forward<br>Reverse	GTTATTAGGGAAGAACATATGTG<br>CCACCTTCCTCCGGTTTGTCACC	750	57	[20]
Candida albicans 18S rRNA[6]	AF114470.1	Forward<br>Reverse	GGATTTACTGAAGACTAACTACTG<br>GAACAACAACCGATCCCTAGT	144	59	[22]
